# Refugee Mental Health—An Urgent Call for Research and Action

**DOI:** 10.1001/jamanetworkopen.2021.2543

**Published:** 2021-03-01

**Authors:** Altaf Saadi, Tala Al-Rousan, Rawan AlHeresh

**Affiliations:** Department of Neurology, Massachusetts General Hospital, Harvard Medical School, Boston (Saadi); Herbert Wertheim School of Public Health and Human Longevity, University of California, San Diego (Al-Rousan); Department of Occupational Therapy, School of Health and Rehabilitation Sciences, MGH Institute of Health Professions, Boston, Massachusetts (AlHeresh).

At the end of 2019, the United Nations (UN) Refugee Agency estimated that there were 26 million refugees worldwide—the highest number ever seen.^1^ This growing exodus of refugees from their home countries is triggered by war, civil unrest, political violence, and other humanitarian crises that fail to realize the human rights of millions of people worldwide. According to the UN Convention Relating to the Status of Refugees, refugees are people forced to flee their home country for fear of persecution based on “race, religion, nationality, membership of a particular social group or political opinion.”^2^

Refugees experience cumulative trauma across their migration journey, including premigratory trauma in their home countries, trauma during their journey to safer areas, and postmigratory trauma involved in the resettlement process. These experiences may involve exposure to violence, sexual assault or rape, human trafficking, unemployment, loneliness, and limited access to food and/or medical care.^3^ Consequently, the prevalence of mental illness, including depression and posttraumatic stress disorder, in this population is high, albeit a systematic review finding that studies reported large variations in mental illness among this population and calling for increased data on this growing and marginalized population.^4^ Notably, studies focus predominantly on discrete aspects of the migration journey (premigratory, migration journey, or postmigratory). The literature is lacking studies that link the continuum of experience. The study by Hossain and colleagues^5^ focusing on a large sample of Rohingya refugees fills this gap.

The Rohingya are a distinct, mostly Muslim ethnic group in Myanmar, ethnically and linguistically distinct from the Burmese majority population. Nearly a quarter of a million Rohingya have been expelled from their homes in Rakhine state, Myanmar, by the Myanmar security forces since August 2017 owing to renewed violence, including rape, murder, and arson. The UN has determined that Myanmar’s security forces showed genocidal intent, and some countries have recognized the crimes committed against the Rohingya as constituting genocide.

Hossain and colleagues^5^ interviewed 1184 Rohingya refugees from 8 refugee camps within Cox’s Bazar, Bangladesh, currently the location of the world’s largest refugee camp. They found that almost half the refugees had severe posttraumatic stress symptoms (PTSSs), and almost one-fourth had probable PTSSs. More than 4 in 10 refugees in the sample reported receiving inadequate humanitarian aid for their families, among whom 57% reported severe PTSSs. Almost 12% of refugees experienced physical and sexual abuse before displacement in Myanmar; 64% of these refugees had severe PTSSs.

The authors found that all types of abuse (verbal, physical, and sexual) were associated with PTSSs. Higher prevalence ratios of PTSSs were associated with increased age, higher rates of predisplacement abuse, less self-reported humanitarian aid, and fewer paid employment opportunities. Stated differently, postmigratory experiences of access to appropriate humanitarian aid and paid employment opportunities reduced the risk of PTSSs. The research approach undertaken by Hossain and colleagues,^5^ associating premigratory and postmigratory realities, is noteworthy given the existing literature gap.

As clinical and public health researchers with expertise in refugee health, we recognize that education, employment, and humanitarian aid represent critical pathways to improve the health of this marginalized population. Opportunities for employment, education, and occupational engagement in meaningful and purposeful daily activities have been shown to reduce mental health symptoms in refugees and other marginalized populations.^5–7^ From an intervention standpoint, these opportunities necessitate interdisciplinary collaboration within and outside of health care. For example, occupational therapists can address personal and contextual barriers to individual engagement in meaningful livelihood activities, thereby improving mental health and quality of life.^8^ Social entrepreneurs or business incubators can partner with refugee-serving agencies to provide employment opportunities that may otherwise be inaccessible to refugees. Advocates and policy makers can promote policy changes that facilitate increased humanitarian aid or increased access to employment opportunities on resettlement.

Despite its many strengths, the article by Hossain and colleagues^5^ must be considered in light of certain limitations. A significant gap in refugee medicine is the lack of cross-cultural validation and accountability of measurement invariance, such that an instrument or questionnaire developed in English is measuring the same construct in other linguistic or cultural groups. This study used the Impact of Event Scale–Revised to determine PTSSs. However, translating questionnaire items from one language to another does not address cultural irrelevancy, potentially jeopardizing measurement precision. For example, emotional distress is explained as the “absence of peace” in the Rohingya culture,^6^ but this concept is not measured in any of the mental health assessments used with Rohingya refugees, leaving significant attributions out of the picture.

In addition, numerous studies aiming to screen and assess refugees’ mental health train their data collectors on consistency and ethics, with a negligible focus on trauma-informed care. Hossain and colleagues^5^ did not provide detailed information about how this training was done. However, they alluded to how the training may have negatively affected their data collection (namely, that they combined physical and sexual abuse when they found that Rohingya women were reluctant to respond to questions about sexual abuse as an individual option on the questionnaires). Building awareness about and implementing trauma-informed refugee research approaches are necessary to avoid retraumatization of refugees and prevent vicarious trauma among data collectors. The roadmap to promoting an evidence base for refugee health must include trauma-informed research approaches.

Last, refugees who live in camps make up about 20% of the entire refugee population. This study should be replicated in other refugee populations to better understand the association of premigratory, migration journey, and postmigratory trauma with mental health outcomes across diverse refugee populations. For medical and public health practitioners motivated to address the needs of refugee populations, the study by Hossain and colleagues^5^ can serve as an important starting point toward policy development and programmatic implementation. In this way, postmigration social factors, such as humanitarian aid or employment opportunities, are not passive circumstances to which refugees are exposed but circumstances borne out of refugee agencies’ and host countries’ operational policy decisions.

Ultimately, we need a robust evidence base paired with carefully planned multidisciplinary programming to effectively and holistically meet the mental health needs of the growing refugee population. This begins with elucidating the nature and effect of premigratory trauma with postmigration social factors using rigorous research studies so that multidisciplinary collaborators, policy makers, and advocates alike can effectively improve refugee populations’ health.
